# Mitochondrial Calcium Increase Induced by RyR1 and IP3R Channel Activation After Membrane Depolarization Regulates Skeletal Muscle Metabolism

**DOI:** 10.3389/fphys.2018.00791

**Published:** 2018-06-25

**Authors:** Alexis R. Díaz-Vegas, Alex Cordova, Denisse Valladares, Paola Llanos, Cecilia Hidalgo, Gaia Gherardi, Diego De Stefani, Cristina Mammucari, Rosario Rizzuto, Ariel Contreras-Ferrat, Enrique Jaimovich

**Affiliations:** ^1^Muscle Physiology Laboratory, Center of Studies in Exercise, Metabolism and Cancer, Institute of Biomedical Sciences, Universidad de Chile, Santiago, Chile; ^2^Biomedical Neuroscience Institute, Center of Studies in Exercise, Metabolism and Cancer, Institute of Biomedical Sciences, Universidad de Chile, Santiago, Chile; ^3^Exercise and Movement Science Laboratory, Universidad Finis Terrae, Santiago, Chile; ^4^Institute for Research in Dental Science, Universidad de Chile, Santiago, Chile; ^5^Department of Biomedical Sciences, University of Padova, Padova, Italy

**Keywords:** energy distribution, inositol 1,4,5-trisphosphate receptor, mitochondria heterogeneity, mitochondrial network, ryanodine receptors

## Abstract

**Aim:** We hypothesize that both type-1 ryanodine receptor (RyR1) and IP_3_-receptor (IP_3_R) calcium channels are necessary for the mitochondrial Ca^2+^ increase caused by membrane depolarization induced by potassium (or by electrical stimulation) of single skeletal muscle fibers; this calcium increase would couple muscle fiber excitation to an increase in metabolic output from mitochondria (excitation-metabolism coupling).

**Methods:** Mitochondria matrix and cytoplasmic Ca^2+^ levels were evaluated in fibers isolated from *flexor digitorium brevis* muscle using plasmids for the expression of a mitochondrial Ca^2+^ sensor (CEPIA3*mt*) or a cytoplasmic Ca^2+^ sensor (RCaMP). The role of intracellular Ca^2+^ channels was evaluated using both specific pharmacological inhibitors (xestospongin B for IP_3_R and Dantrolene for RyR1) and a genetic approach (shIP_3_R1-RFP). O_2_ consumption was detected using Seahorse Extracellular Flux Analyzer.

**Results:** In isolated muscle fibers cell membrane depolarization increased both cytoplasmic and mitochondrial Ca^2+^ levels. Mitochondrial Ca^2+^ uptake required functional inositol IP_3_R and RyR1 channels. Inhibition of either channel decreased basal O_2_ consumption rate but only RyR1 inhibition decreased ATP-linked O_2_ consumption. Cell membrane depolarization-induced Ca^2+^ signals in sub-sarcolemmal mitochondria were accompanied by a reduction in mitochondrial membrane potential; Ca^2+^ signals propagated toward intermyofibrillar mitochondria, which displayed increased membrane potential. These results are compatible with slow, Ca^2+^-dependent propagation of mitochondrial membrane potential from the surface toward the center of the fiber.

**Conclusion:** Ca^2+^-dependent changes in mitochondrial membrane potential have different kinetics in the surface vs. the center of the fiber; these differences are likely to play a critical role in the control of mitochondrial metabolism, both at rest and after membrane depolarization as part of an “excitation-metabolism” coupling process in skeletal muscle fibers.

## Introduction

The skeletal muscle system plays a critical role in the energy balance of the organism. During contraction, its energy requirements can increase nearly instantaneously by more than 100-fold (Weibel and Hoppeler, 2005). This skeletal muscle characteristic suggests the need for a fast signaling mechanism responsible for the metabolic increase generated by muscle plasma membrane depolarization. We define as “excitation-metabolism coupling” this functional interaction between plasma membrane depolarization and the resulting increase in muscle metabolism.

Mitochondrial function and other bioenergetics pathways such as glycolysis are likely to play critical roles in maintaining the energy balance between supply and demand. Moreover, mitochondrial dysfunction characterizes a wide spectrum of adult-onset degenerative diseases, including muscle atrophy, muscle dystrophy, insulin resistance, type 2 diabetes, age-related sarcopenia, among others (Arnould et al., 2015), which may affect the excitation-metabolism coupling process.

It is well known that mitochondrial metabolism is the main source of cellular ATP generation in several cell types and that mitochondrial activity increases upon Ca^2+^ accumulation in mitochondria (Murgia and Rizzuto, 2015). The flow of electrons along the electron transport chain results in the translocation of H^+^ ions across the inner mitochondrial membrane (IMM) from the matrix to the intermembrane space (IMS). This process contributes to create a large mitochondrial membrane potential (ΔΨmt) that, besides its key role in ATP synthesis, produces a large driving force for Ca^2+^ uptake into the mitochondrial matrix (Mitchell, 1961). Interestingly, plasma membrane depolarization of muscle fibers increases mitochondrial Ca^2+^ levels (Mammucari et al., 2015), leading to the activation of several Ca^2+^-sensitive dehydrogenases of the Krebs cycle, and providing increased substrate availability for the electron transport chain (Das and Harris, 1990).

Mitochondrial Ca^2+^ uptake involves the passage of Ca^2+^ through the ion-impermeable IMM by means of the mitochondrial Ca^2+^ uniporter (MCU) complex, recently identified and characterized (Baughman et al., 2011; De Stefani et al., 2011). Interestingly, MCU^−/−^ mice exhibit impaired ability to perform physical exercise and lack mitochondrial Ca^2+^ uptake (Pan et al., 2013). Although a previous report proposed that the mitochondrial [Ca^2+^] increase provoked by muscle fiber depolarization is sufficient to augment mitochondrial metabolism (Dirksen, 2009), there is no direct evidence in adult skeletal muscle fibers to substantiate this mechanism with the use of appropriate experimental tools. Hence, a re-examination of the role of mitochondrial Ca^2+^ levels on mitochondrial metabolism immediately after depolarization of adult skeletal muscle fibers is needed.

Skeletal muscle activity requires constant mitochondrial ATP production; this is a process with a very high-energy cost since the differences in energy demand between resting and contracting muscle are considerable (Weibel and Hoppeler, 2005). In addition to metabolite availability, a fast mechanism to couple muscle activation to mitochondrial metabolism appears essential to maintain the balance between ATP demand and supply. In fact, disruption of mitochondrial integrity can lead to a reduction in muscle function and compromised cell bioenergetics (Primeau et al., 2002).

Plasma membrane depolarization induces two different Ca^2+^ transients in skeletal muscle cells; the first transient, which is part of the excitation-contraction coupling (ECC) process, represents a fast [Ca^2+^] rise that emerges from Ca^2+^ release through ryanodine receptor type-1 (RyR1) Ca^2+^ channels. The second Ca^2+^ transient is slower and depends on activation of the inositol 1,4,5-trisphosphate receptor (IP_3_R); this slow transient relates to gene expression and mediates the excitation-transcription coupling (ETC) process (Jaimovich et al., 2000; Araya et al., 2003; Bustamante et al., 2014). The idea that Ca^2+^ released via IP_3_R present in the sarco-endoplasmic reticulum (SER) is amenable to transfer to the mitochondria has been proposed in other cell types (Bravo-Sagua et al., 2013). In addition, knockdown of mitofusin1 reduces the SER-mitochondria interaction in skeletal muscle fibers, resulting in a decrease of mitochondrial Ca^2+^ uptake after depolarization (Ainbinder et al., 2015). Nevertheless, the role of the intracellular Ca^2+^ channels, IP_3_R and RyR1, in this process is still unresolved. Accordingly, we explored here whether Ca^2+^ release mediated by IP_3_R/RyR1 channels mediates the metabolic change induced by surface cell membrane depolarization, and further explored the underlying signaling mechanism. We have to take into account that little attention has been paid so far to the role of RyR1 and IP_3_R on mitochondrial Ca^2+^ increase and mitochondrial metabolism after depolarization in skeletal muscle.

The contribution of mitochondrial metabolism to muscle function is well accepted. Nonetheless, the mechanism by which the H^+^ electrochemical gradient generated by oxidative phosphorylation distributes within mitochondria present in different regions of the skeletal muscle fiber is completely unknown. The pathways proposed until now to facilitate the diffusion of both ATP and ADP within the cell, and O_2_ diffusion from blood to mitochondria, are the creatine kinase shuttle system and the oxy-deoxy myoglobin shuttle, respectively (Wittenberg, 1970; Bessman and Geiger, 1981). However, mice that lack myoglobin (Garry et al., 1998), creatine kinase (Kernec et al., 2001) or creatine (Lygate et al., 2013) do not show altered skeletal muscle performance. Recently, Glancy et al. (2015) demonstrated that adult skeletal muscle fibers possess a complex and interconnected network of mitochondria that extends from the subsarcolemmal to the intermyofibrillar region. Interestingly, these authors showed that flash release of a caged mitochondrial uncoupler in a very small volume deep into the muscle fiber causes a fast and coordinated depolarization of ΔΨmt throughout the fiber (Wittenberg, 1970). Altogether, these results suggest that mitochondria are interconnected, creating a functional organelle that by allowing differential regulation of the H^+^ gradient, could promote energy production in specific subcellular locations of the muscle fiber. Nevertheless, a mechanism responsible for triggering rapid changes in ΔΨmt in response to physiological stimuli in skeletal muscle fibers has not been described.

The aim of the this work was to test the hypothesis that plasma membrane depolarization increases mitochondrial Ca^2+^ levels by a mechanism dependent on IP_3_R/RyR1 activation, leading to an increase of mitochondrial metabolism in adult muscle fibers in a process named “Excitation-Metabolism Coupling.” This process includes ΔΨmt transfer between neighboring mitochondria from the subsarcolemmal to the intermiofibrillar regions as a mechanism that might participate in energy administration in response to the subcellular energy demands of adult skeletal muscle fibers.

## Results

### Plasma membrane depolarization induced by high K^+^ enhances the rate of oxygen consumption in skeletal muscle fibers

**Figure 1:**
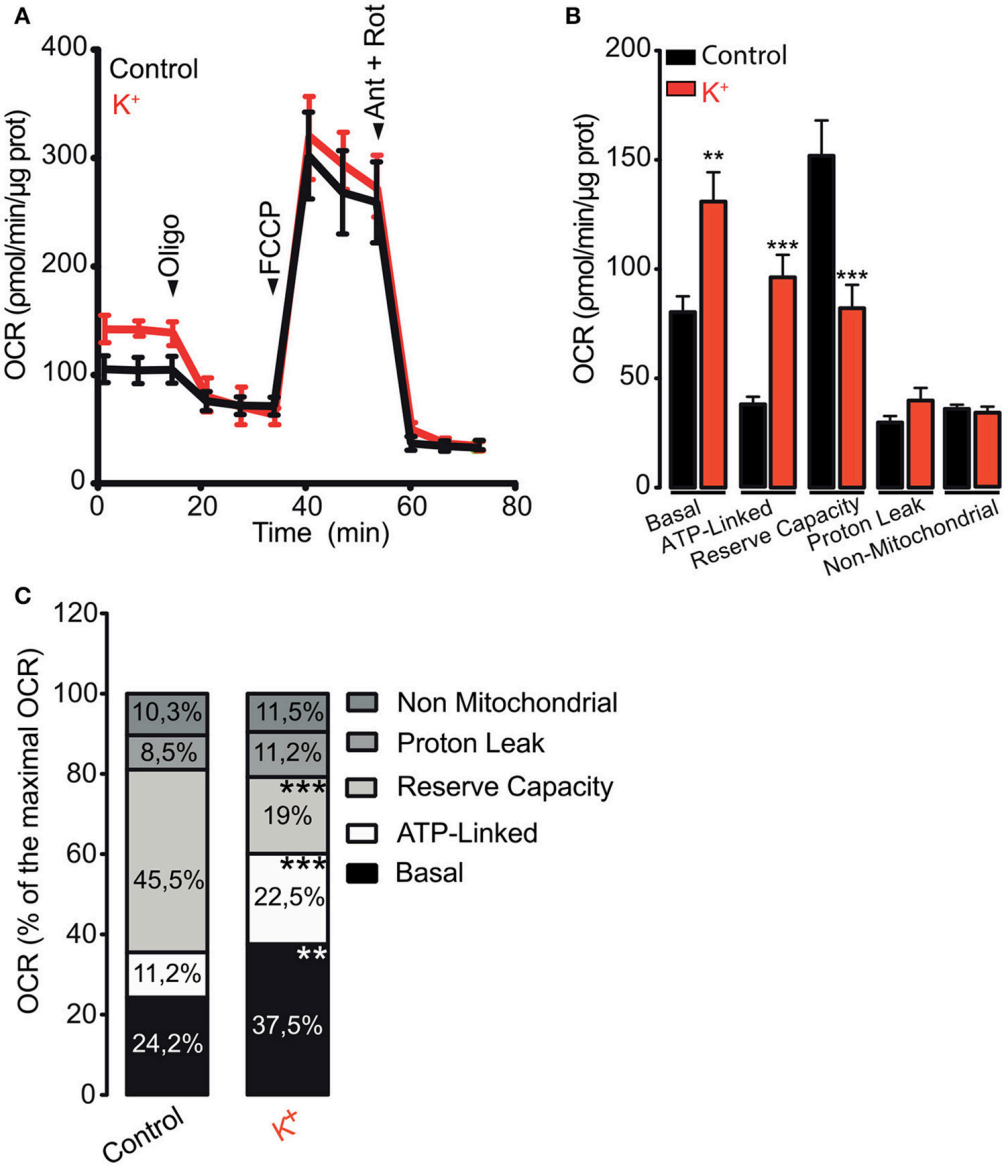
Membrane depolarization promotes oxygen consumption ratio in skeletal muscle fibers. Muscle fibers were isolated from FDB muscle and cultured overnight; fibers were depolarized using 65 mM K^+^ during 5 min before measurement started. **(A)** Representative kinetics of OCR in control or depolarized muscle fibers. **(B)** Quantification of the basal, ATP-linked, reserve capacity, proton leak and non-mitochondrial OCR were determined as described in section Materials and Methods. **(C)** Percent distribution of the basal, ATP-linked, reserve capacity, proton leak and non-mitochondrial OCR were calculated. OCR after FCCP administration was considered as 100% (maximal OCR). *N* = 5 different animals ^**^p < 0.01; ^***^p < 0.001 compared with the control condition.

The energy requirements of the skeletal muscle tissue increase during contraction (Weibel and Hoppeler, 2005). However, the specific effects of muscle activation on the oxygen consumption rate (OCR) of isolated adult skeletal muscle fibers are poorly understood. In order to evaluate the OCR on adult muscle fibers, we depolarized the fibers using a high K^+^ medium before the OCR assay. Cell membrane depolarization increased the basal OCR from 82.1 ± 7.1 to 130.9 ± 13.5 pmol/min/μg protein (Figures 1A,B), corresponding to 24.2 and 37.5% of the maximal OCR respectively (Figure 1C). Additionally, depolarization augmented the OCR linked to ATP synthesis (OCR ATP-Linked) from 38.2 ± 3.4 to 78.5 ± 12.6 pmol/min/μg protein (Figures 1A,B), which corresponds to 11.2 and 22.5% of the maximal OCR respectively (Figure 1C). In accord with the increase in basal OCR, depolarization reduced the maximal reserve, from 154.5 ± 16.2 to 66.7 ± 10.7 pmol/min/μg protein (45.5 and 19% of the maximal OCR respectively) (Figures 1A–C). Interestingly, K^+^-induced depolarization of muscle fibers did not affect the maximal respiratory capacity, the proton leak ($L_{{\text{H}}^{+}}$) or the non-mitochondrial OCR (Figures 1A–C). These results suggest that K^+^-induced membrane depolarization directly stimulates mitochondrial metabolism in skeletal muscle fibers.

### Mitochondrial Ca^2+^ levels visualized with CEPIA3*mt*

**Figure 2:**
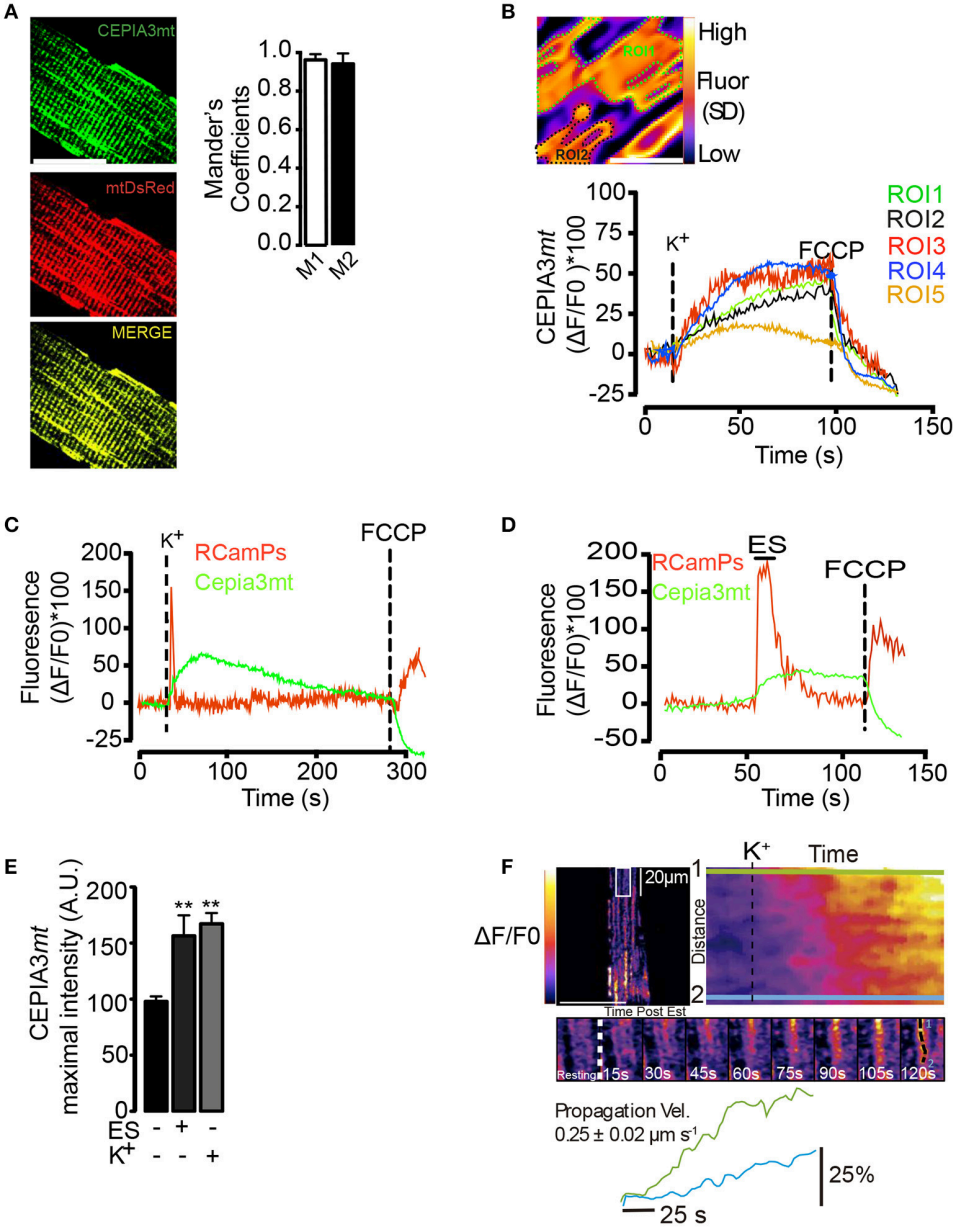
Mitochondrial Ca^2+^ levels visualized with CEPIA3*mt*. Adult FDB muscles were electroporated with plasmids encoding the molecular Ca^2+^ sensor CEPIA3*mt* and/or RCaMPs directed to mitochondria and cytoplasmic compartment, respectively. **(A)** Muscle fibers were co-electroporated with plasmids encoding CEPIA3mt (Left top panel) mtDsRed (left middle panel). High co-localization was observed between both sensors (Left bottom panel). Mander's coefficient were evaluated (right panels), scale bar is 15 μm. **(B)** Standard deviation of fluorescence, representative ROI (ROI1 and ROI2) selected by fiber and representative kinetics of 5 different ROIs are shown. Scale bar is 2 μm. **(C)** The fibers expressing CEPIA3*mt* (green line) plus RCaMPs (red line) were stimulated with 65 mM K^+^; FCCP (1 μM) was added at the end of experiments to uncouple mitochondria. Mean of 5 different animals is shown. C) FDB fibers electroporated with plasmids encoding CEPIA3*mt* (green line) and RCamPs (red line) were exposed to electrical stimulation; representative kinetics and the maximal fluorescence obtained is shown **(D,E)**. **(F)** Intramitochondrial calcium wave propagation in fibers expressing CEPIA3mt stimulated with 65 mM K^+^. *N* = 4 different animals and 25 fibers were evaluated in each case. ^**^p < 0.01 vs. control.

An increase in mitochondrial Ca^2+^ levels (Mitchell, 1961) activates different Ca^2+^-sensitive dehydrogenases of the Krebs cycle (Das and Harris, 1990) and enhances, in consequence, ATP synthesis and mitochondrial O_2_ consumption. Mitochondrial Ca^2+^ increases in response to different stimuli have been shown in different cell models using both fluorescent dyes and molecular tools. In order to evaluate the mitochondrial Ca^2+^ levels in muscle fibers, we used the low affinity indicator (Kd 11 μM) CEPIA3*mt* (Ca^2+^-measuring protein indicator type 3). We electroporated adult FDB muscles *in vivo* with a plasmid that promotes the expression of the CEPIA3*mt* sensor protein with selective mitochondrial destination (Suzuki et al., 2014). CEPIA3*mt* exhibited a high co-localization with mtDsREd (Figure 2A). Mander's coefficient analysis (Figure 2A, right panel) showed high coincidence between CEPIA3*mt* and mtDsREd. Confocal images displayed over 95% of co-localization, strongly suggesting mitochondrial compartmentalization of the molecular CEPIA3*mt* indicator.

The selected region of interest (ROI) was determined after the projection of the standard deviation of fluorescence over time (Figure 2B). Membrane depolarization with high K^+^ solution induced a slow and sustained increase of mitochondrial Ca^2+^ levels, which reached maximal fluorescence 30 s (± 5 s) after stimulation; the addition of 0.5 μM FCCP (Carbonyl cyanide-p-trifluoromethoxyphenylhydrazone) caused a similar dissipation of this increase in both selected ROIs (Figure 2B, right panel) (Movie S1). Similar mitochondrial Ca^2+^ changes were observed in 25 selected ROIs from one fiber (Figure 2B). The maximal change in fluorescence was 51% (±10%) (Figure 2E). To ensure that the mitochondrial Ca^2+^ increase did not arise from a spurious effect of high K^+^, we co-electroporated plasmids to express jointly the red cytoplasmic Ca^2+^ indicator GECI (RCaMP) and CEPIA3*mt* and subjected the isolated fibers to electrical stimulation (ES). As shown in Figure 2C, ES increased both cytoplasmic and mitochondrial Ca^2+^ signals. Exposure to the high K^+^ solution induced a similar increase in RCamPs and Cepia3mt fluorescence (Figure 2D); likewise, exposure to the high K^+^ solution or ES produced similar maximal fluorescence increases (Figure 2E). The time courses measured at two subcellular locations indicated intramitochondrial Ca^2+^ wave-like propagation in the subsarcolemmal region of the fibers, spanning the whole area of the confocal slice (Figure 2F, upper and middle panels) (Movie S2). The speed of these waves was 0.25 ± 0.02 μm/s (Figure 2F, bottom panel). The Ca^2+^-dependent Cepia3*mt* signal was sensitive to inhibition by Ruthenium Red (Figure S5), suggesting that this signal is indeed due to mitochondrial Ca^2+^ uptake via the MCU.

In order to determine if exposure to 65 mM K^+^ during 1 min induced muscle fiber damage, we performed a control on the excitability of the fiber. To this aim, muscle fibers loaded with Fluo4-AM were exposed to electrical stimulation (ES) or high K^+^ medium. As shown in Figure S1, ES induced a fast cytoplasmic transient. One minute exposure to the high K^+^ medium induced a smaller and longer (≈10 s) calcium transient compared to ES. 40 s after removal of the high K^+^ medium, the muscle fibers recovered their response to ES suggesting that treatment with high K^+^ (65 mM during 1 min) did not affect the long term viability of the muscle fibers (Figure S1).

### Activation of both IP_3_R and RyR1 is required for mitochondrial Ca^2+^ increase induced by plasma membrane depolarization by high potassium

**Figure 3:**
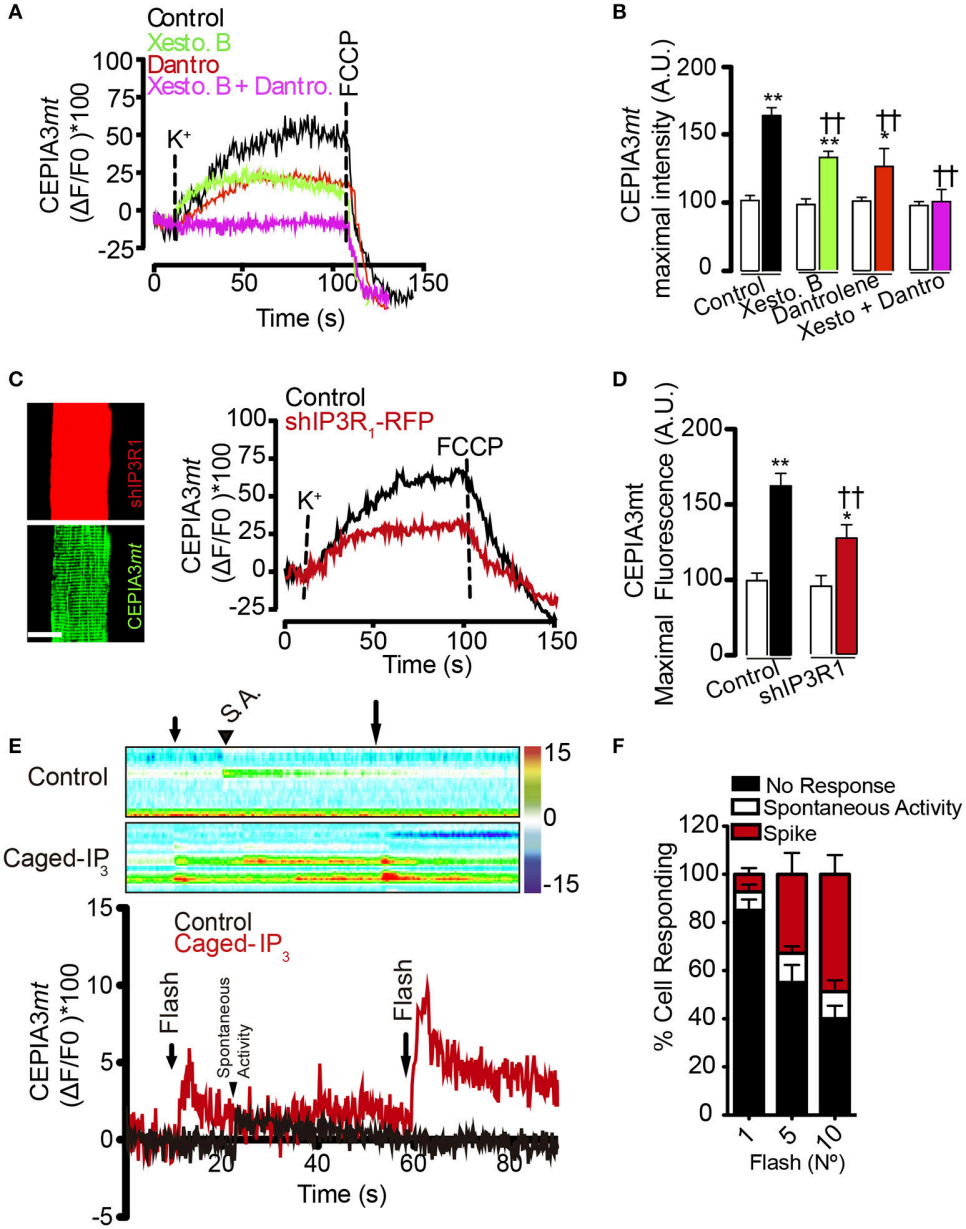
Activation of IP_3_R and RyR1 participate in the mitochondrial Ca^2+^ increase after membrane depolarization. Adult FDB muscle was electroporated with plasmids encoding the molecular Ca^2+^ sensor CEPIA3*mt*. Fibers were maintained in Krebs Ringer buffer with BTS (10 μM) and pre-incubated for 1 h with xestospongin B (10 μM), dantrolene (50 μM) or both. The representative kinetics and maximal fluorescence was calculated in each condition **(A,B)**. sh-IP_3_R1-RFP partially reduced the calcium increases in response to high K^+^ **(C,D)**, scale bar is 15 μm. **(E)** Muscle fibers electroporated with plasmids encoding CEPIA3mt and pre-incubated with caged IP_3_. The photolysis increased the mitochondrial calcium level. Color bar represents the relative change in fluorescence. **(F)** Percent of response to different number of UV flashes is shown. *N* = 6 experiments were performed and 25 fibers were evaluated each time. †Means difference vs. control stimulated with K^+^. ^*^Difference vs. resting; ^*^p < 0.05; ^**/††^*p* < 0.01.

The two main intracellular Ca^2+^ channels in the skeletal muscle fiber are RyR1 and IP_3_R channels. In order to address if these intracellular Ca^2+^ channels are involved in the mitochondrial Ca^2+^ increases induced by high K^+^-induced depolarization, we used both pharmacological and genetic approaches. We observed that the depolarization-dependent mitochondrial Ca^2+^ increase was partly prevented by either dantrolene (50 μM) or xestospongin B (10 μM). The maximal fluorescence observed was 66.2% (±8.0) in the control, 29.5% (±1.3) in dantrolene pre-treated fibers and 33.0% (±1.9) in xestospongin B pre-treated cells (Figure 3A). Furthermore, the mitochondrial Ca^2+^ increase induced by depolarization was completely suppressed when using both inhibitors simultaneously (Figure 3A), (quantification of maximal fluorescence is shown in Figure 3B). Finally, dantrolene but not xestospongin B reduced the slope of the fluorescence increase from 0.55 ± 0.01 to 0.15 ± 0.12 (dF/dT ^*^ s^−1^). These results are consistent with the kinetics of activation of RyR1 (fast) and IP_3_R (slow) in muscle fibers. Furthermore, these findings suggest that Ca^2+^ release through both IP_3_R and RyR1 are involved in the mitochondrial Ca^2+^ increase produced by depolarizing stimuli.

In order to corroborate the contribution of IP_3_R to mitochondrial Ca^2+^ levels, we used fibers co-electroporated with plasmids to express CEPIA3*mt* and shIP_3_R1-RFP, since IP_3_R1 is the main IP_3_R isoform expressed in the adult muscle fiber (Powell et al., 2001). Electroporation with shIP_3_R1-RFP reduced the IP_3_R1 level by 80%, approximately (Figure S2). In line with the pharmacological approach, the mitochondrial Ca^2+^ increase observed after depolarization with high K^+^ was partially reduced in fibers containing shIP_3_R1-RFP compared to the control; the maximal fluorescence increase was 61% and 26.9% in control and shIP_3_R1-RFP transfected fibers, respectively (Figures 3C,D). Additionally, fibers expressing CEPIA3*mt* pre-incubated with caged-IP_3_ showed a 13% (±3.0%) increase in mitochondrial Ca^2+^ levels after each photo-release (10 flashes) of IP_3_ compared with the control (Figures 3E,F). The amplitude of the Ca^2+^ signals, and the percentage of cells responding, increased proportionally with the number of UV flashes applied (1 flash; 7.3%; 5 flashes; 32.3%; 10 flashes; 48.8% of the total cells analyzed responded to the stimulus).

Altogether, these findings indicate that activation of both IP_3_R1- and RyR1-mediated Ca^2+^ release contribute to the mitochondrial Ca^2+^ increase, and thus to the mitochondrial functional activation triggered by muscle stimulation (and contractile activity).

### Activation of RyR1 but not IP_3_R is necessary to increase the mitochondrial O_2_ consumption after plasma membrane depolarization

**Figure 4:**
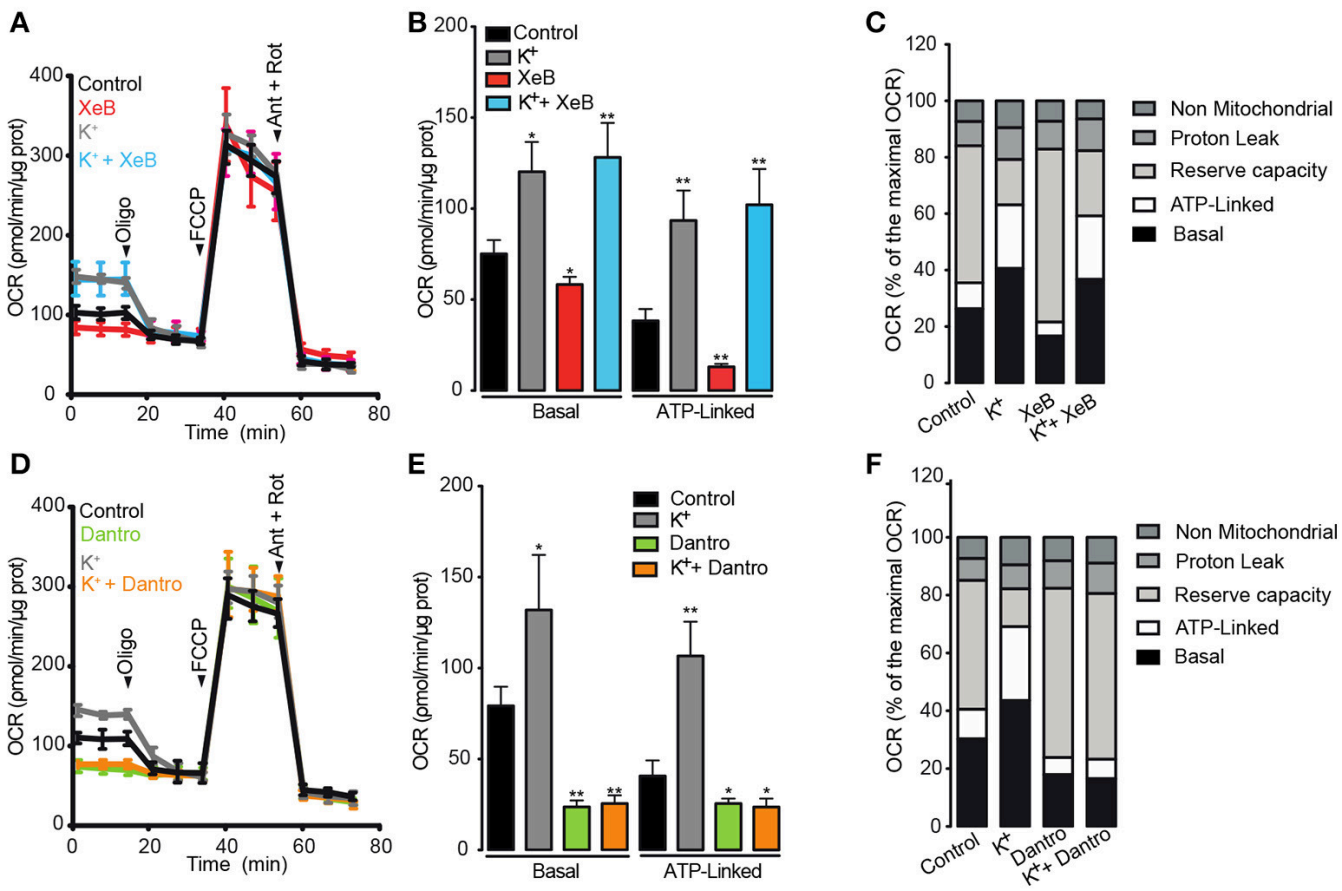
Activation of RyR1 but not of IP_3_R is necessary to increase the mitochondrial O_2_ consumption after membrane depolarization. Adult FDB muscle fibers maintained in Seahorse medium with BTS (10 μM) were pre-incubated for 1 h with xestospongin B (10 μM) or dantrolene (50 μM). The fibers were depolarized 5 min before the measurement. **(A,D)** Representative kinetics of OCR in fibers pre-incubated with xestospongin B or dantrolene respectively. **(B,E)** Quantification of basal and ATP-linked OCR in muscle fibers pre-incubated with xestospongin B or dantrolene respectively. **(C,F)** Percent distribution of the basal, ATP-linked, reserve capacity, proton leak and non-mitochondrial OCR were calculated. OCR after FCCP administration was considered as 100% (maximal OCR). *N* = 5 independents experiments. ^*^p < 0.05; ^**^p < 0.01 compared with the control condition.

According to the results detailed above, we hypothesized that both IP_3_R and RyR1 are necessary to induce the metabolic increase caused by membrane depolarization. To test this hypothesis, OCR was evaluated after depolarization in muscle fibers pre-incubated with the specific inhibitors xestospongin B and dantrolene. We first focused on the role of IP_3_R on membrane depolarization-dependent mitochondrial OCR increase. As previously shown, depolarization with high K^+^ increased both, the basal and the ATP-linked OCR (Figures 4A–C). Compared to the control, the basal OCR was partly reduced by xestospongin B; the OCR was 80.1 ± 3.2 pmol/min/μg for control and 64.0 ± 6.3 pmol/min/μg for xestospongin B-treated fibers, corresponding to 22.2 and 16.6% of the maximal OCR respectively. The ATP-linked OCR was also reduced from 39.5 ± 4.2 to 27.4 ± 4.2 pmol/min/μg in cells pre-incubated with xestospongin B; these values correspond to 11.8 and 7.0% of the maximal OCR. Additionally, we did not observe differences in the maximal OCR, the *L*_H_+, or the non-mitochondrial OCR following the addition of xestospongin B. Moreover, xestospongin B did not prevent the basal or the ATP-linked OCR increase induced by depolarization (Figures 4A–C).

We subsequently investigated the role of RyR1 on the membrane depolarization-dependent mitochondrial OCR increase. As observed with xestospongin B, dantrolene partly reduced both the basal and the ATP-linked OCR. The basal OCR was reduced from 83.2 ± 1.2 pmol/min/μg to 66.1 ± 5.7 pmol/min/μg in cells pre-incubated with dantrolene, corresponding to 25.3 and 17.8% of the maximal OCR, respectively (Figures 4D–F). In addition, dantrolene also reduced the ATP-linked OCR from 40.1 ± 3.9 to 22.6 ± 3.2 pmol/min/μg (Figures 4D,E); these values correspond to 11.7 and 5.9%, respectively, of maximal OCR (Figure 4F). Dantrolene did not affect the maximal, the $L_{{\text{H}}^{+}}$ or the non-mitochondrial OCR (Figures 4D–F). The basal and ATP-linked OCR increase caused by depolarization was completely prevented in fibers pre-treated with dantrolene, without effect on the maximal OCR, the $L_{{\text{H}}^{+}}$ or the non-mitochondrial OCR (Figures 4D–F). Altogether, these results strongly suggest that both intracellular Ca^2+^ channels are involved in maintaining the basal and ATP-linked OCR at resting condition. Moreover, the activation of RyR1 but not IP_3_R is necessary for membrane depolarization-dependent O_2_ consumption.

### Heterogeneous distribution of intra-mitochondrial proteins and Ca^2+^ handling between SSM and IMFM in FDB muscle

The mitochondrial Ca^2+^ uptake depends mainly on ΔΨmt and MCU complex distribution (Mitchell, 1961). As muscle fibers possess different types of mitochondria (Picard et al., 2013), we tested the distribution of proteins belonging to the electron transport chain subcomponents and the MCU complex between these two mitochondrial populations in muscle fibers.

**Figure 5:**
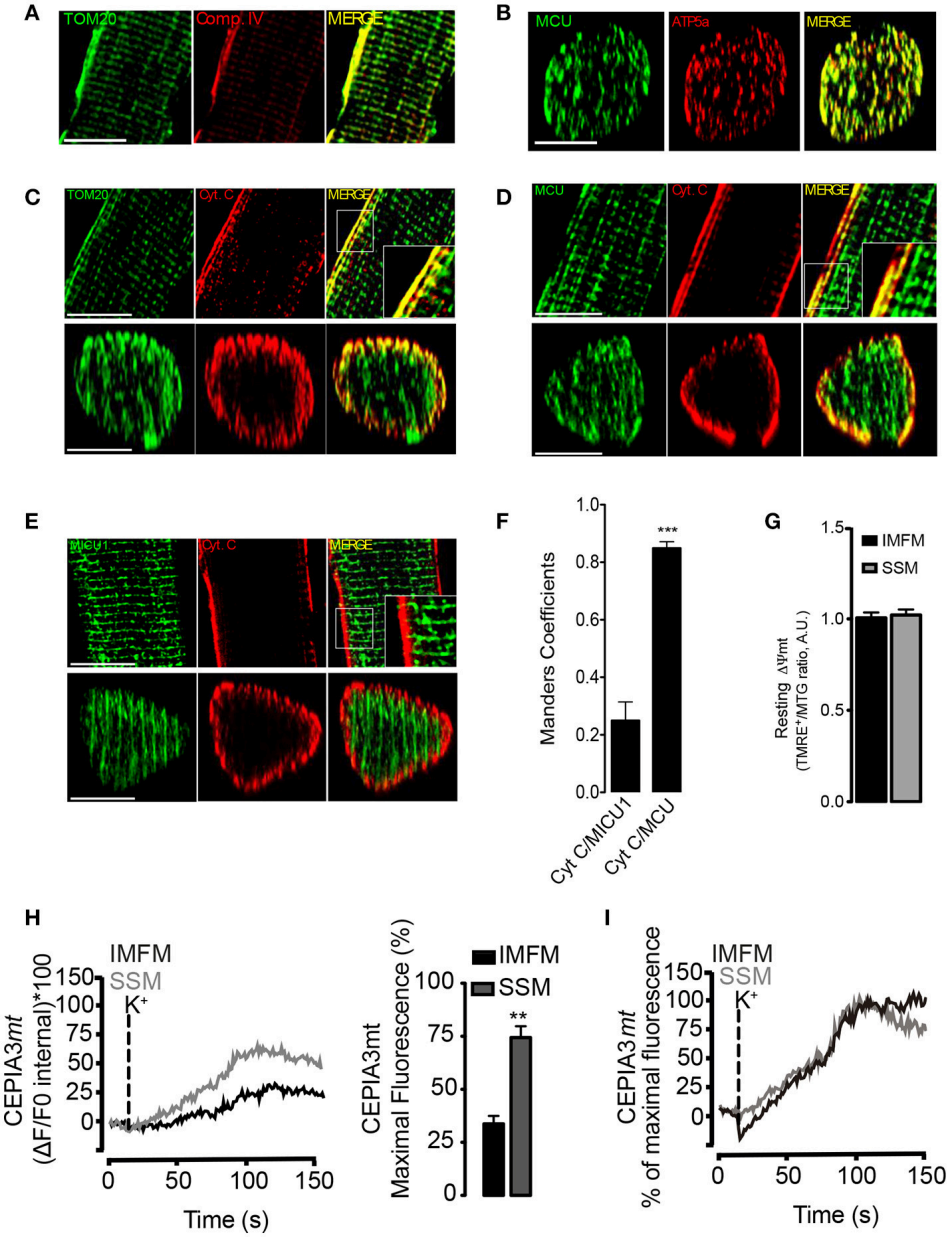
Heterogeneity in intramitochondrial proteins and calcium handling between SSM and IMFM in FDB fibers. **(A)** Immunostaining of complex IV and TOM20 in 1 μm confocal slices of adult muscle fibers, scale bar is 15 μm. **(B)** 3D reconstruction of immunostaining of MCU and ATP5a in adult muscle fibers, scale bar is 15 μm. **(C)** Immunostaining of Cytochrome C (CytC) and TOM20 in 1 μm confocal slices (upper panel). The Z-projection reconstruction of the whole fiber is shown (bottom panel), scale bar is 15 μm. **(D)** Inmunostaining of MCU plus CytC. The longitudinal or z-projection reconstruction is shown in the upper and bottom panel, respectively, scale bar is 15 μm. **(E)** Inmunostaining of MICU1 plus CytC. The longitudinal or z-projection reconstruction is shown in the upper and bottom panel respectively, scale bar is 15 μm. **(F)** Mander's coefficient for Cyt C/MICU1 and Cyt C/MCU. **(G)** Muscle fibers isolated from FDB muscle were incubated during 30 min with TMRE^+^ plus mitotracker green (MTG). One optical slice at 2 μm from the center of the fiber is shown. The ratio of TMRE^+^/MTG fluorescence quantification is shown. **(H)** Adult FDB muscle expressing the molecular Ca^2+^ sensor CEPIA3*mt* were maintained in Krebs Ringer buffer with BTS (10 μM). The change of fluorescence in the *x,z,t* axis was evaluated. Representative kinetics from the subsarcolemmal area (first 5 μm) or the intermyofibrillar area was determined (Left panel) and maximal fluorescence was calculated in the subsarcolemmal or intermyofibrillar area (right panel). **(I)** Maximal fluorescence of SSM or IMFM was adjusted as 100% in order to evaluate the kinetic of fluorescence increase. There is no difference in the slope of calcium increase between SSM and IMFM. *N* = 6 different animals and 25 fibers were evaluated in each case. ^**^p < 0.01 vs. IMFM. ^***^p < 0.001 difference vs Cyt/MICU1.

Recently, Glancy et al. demonstrated that complex IV of the ETC was mainly located in the periphery of the muscle fiber (Glancy et al., 2015). In agreement with Glancy et al., in this work we found that complex IV was enriched in the periphery of the fiber, while the structural protein TOM20 had a homogeneous distribution through the fibers (Figure 5A). In order to determine if the observed distribution of complex IV may be due to an antibody penetration problem associated to the co-immunofluorescence, we decided to evaluate the subcellular distribution of MCU plus ATP5a. As shown in Figure 5B, both proteins exhibited a homogenous distribution through the fiber. These results suggest that the differential distribution of complex IV is not associated with antibody penetration problems. Additionally, immunofluorescence assays of Cytochrome C (CytC), another protein of the electron transport chain, showed a strong co-localization with TOM20 only in the sub-sarcolemmal regions; CytC immunostaining showed preponderant fluorescence intensity near the fiber surface, similar to complex IV distribution (Figure 5C, Movie S4).

Using CytC as SSM marker, we also evaluated the distribution of MCU and MICU1 proteins. We found that MCU presented a homogenous distribution within the cell (Figure 5D) with a high Mander's coefficient with CytC (CytC/MCU) in the surface of the cell (0.82 ± 0.05) (Figure 5F), whereas MICU1 was distributed only in the IMFM and was excluded from the subsarcolemmal regions (Figure 5E), with a low Mander's coefficient (0.22 ± 0.08) with CytC (CytC/MICU1) in this region (Figure 5F). These results were consistent with a 3D reconstruction of the fibers (Movies S5, S6). Anti-body against MICU1 was validated in the whole lysate from WT or MICU1 KO MED cells (Figure S3).

Considering the heterogeneous distribution of the ETC and MCU complex we measured the functional parameters ΔΨmt and Ca^2+^ handling respectively. We did not find differences in ΔΨmt values between SSM and IMFM in resting condition (Figure 5G). Membrane depolarization with high K^+^ induced a sustained mitochondrial Ca^2+^ increase in both SSM and IMFM. Even when MCU distribution was homogeneous within the muscle fiber (Figure 5D), the mitochondrial calcium increase in the IMFM exhibited a delay (20 ± 2 s) compared to SSM (Figure 5H). When the maximal fluorescence reached by IMFM and SSM was adjusted to 100%, no differences in the slope of fluorescence increase were observed (Figure 5I).The maximal fluorescence was 55 ± 4% in the SSM and 27 ± 3% in the IMFM (Figure 5H, right panel) (Movie S3).

### Plasma membrane depolarization induced heterogeneous Δψmt and Ca^2+^ handling in SSM and IMFM in FDB muscle

**Figure 6:**
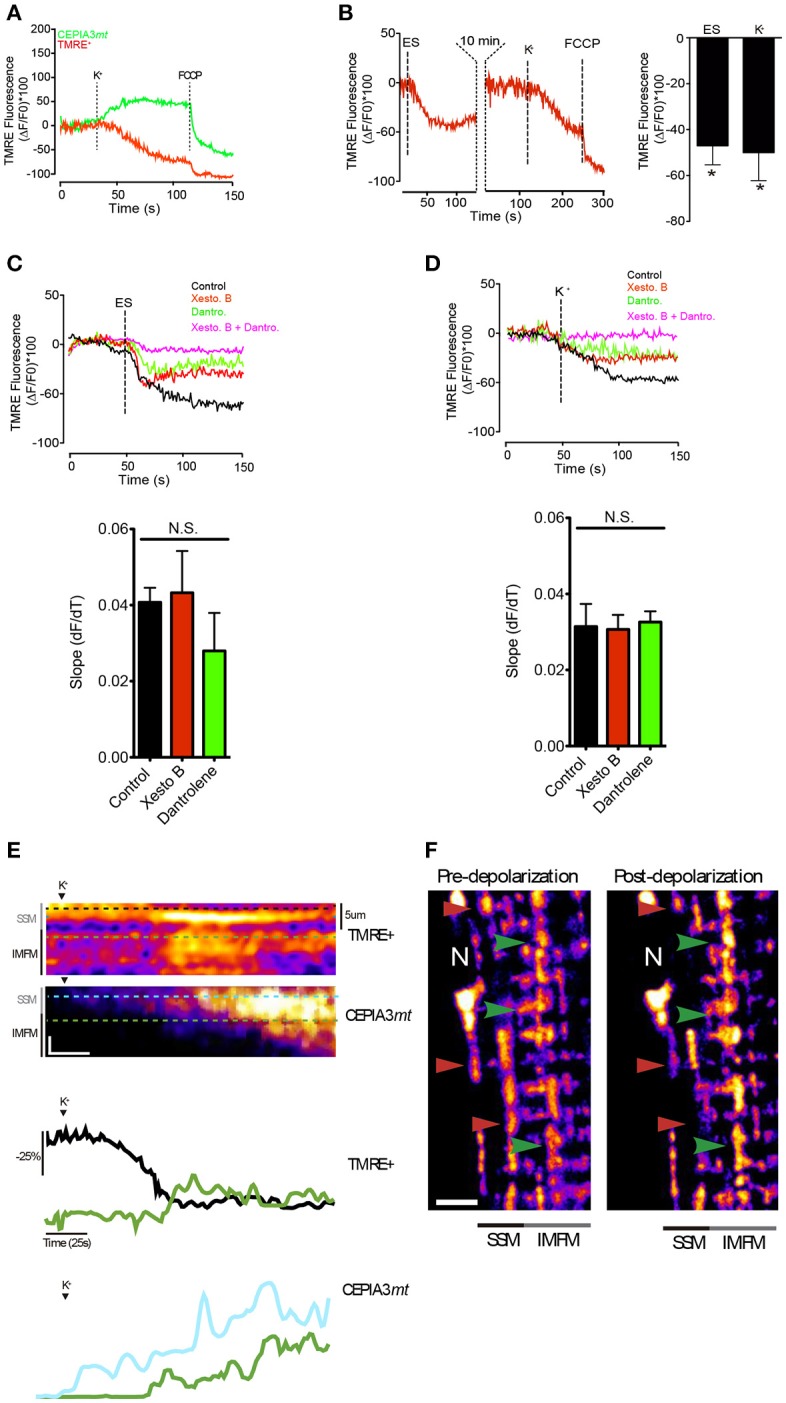
ΔΨm appearsto propagate away from the surface regions toward the central zone. **(A)** Muscle fibers electroporated with plasmids encoding CEPIA3mt were incubated with TMRE+ (20 nM) during 30 min. The subsarcolemmal mitochondrial fluorescence was measured after depolarization with high K^+^ medium. Representative kinetics is shown. **(B)** Muscle fibers were incubated with TMRE^+^ (20 nM) and the fluorescence in the subsarcolemmal region was evaluated after electrical stimulation or high K^+^ medium. At the end of the experiment 0.5 μM FCCP was added to depolarize the mitochondria (left panel). The quantification of the fluorescence is shown in the right panel. **(C)** Muscle fibers incubated with TMRE^+^ (20 nM) were stimulated with ES in presence of different inhibitors. Slope of mitochondrial depolarization is shown (botton panel). **(D)** Muscle fibers incubated with TMRE^+^ (20 nM) were stimulated with high K^+^ medium in presence of different inhibitors. Slope of mitochondrial depolarization is shown (botton panel). **(E)** Line scan of the subsarcolemmal and intermyofibrillar region was performed after K^+^-induced depolarization; the representative image of ΔΨm change and calcium handling (upper panel) and kinetics (bottom panel) are shown. Vertical scale bar represents 10% fluorescence change of. Horizontal scale bar corresponds to 25 s. **(F)** Muscle fibers were incubated with TMRE+ (20 nM) and the images were acquired before and 60 s after potassium depolarization. Red arrowhead shows reduction of fluorescence near the surface of the fiber and green arrowhead shows areas of increase in fluorescence toward the center of the fiber. Scale bar 5 μm. *N* = 6 different animals and 20–25 fibers were analyzed in each condition. ^*^p < 0.05.

Previous image analysis demonstrated that the SSM are morphologically continuous to most IMFM in muscle fibers (Glancy et al., 2015). This feature would provide a putative conductive pathway through the mitochondria intermembrane space. In addition, a previous study showed that Ca^2+^ influx into the mitochondria depolarizes the IMM, reducing ΔΨmt and thus blunting its own driving force (Wacquier et al., 2016). We hypothesized that a depolarization-dependent Ca^2+^ uptake in the SSM stimulates an increase of ΔΨmt in the IMFM, where it is required to maintain *in situ* sarcomeric ATP synthesis. In order to test this hypothesis, muscle fibers expressing CEPIA3*mt* were incubated with TMRE^+^ (Tetramethylrhodamine, Ethyl Ester, Perchlorate) in a non-quenching mode. As shown in Figure 6A, membrane depolarization transiently decreased ΔΨmt in the SSM region, with a maximal effect at 60 s (±3 s) after the stimulus; this decrease correlated with the time-course of the augmented mitochondrial Ca^2+^ levels evaluated by CEPIA3*mt*; ΔΨmt reduction exhibited a delay (10–15 s) compared to the mitochondrial Ca^2+^ increase (Movies S7, S8). According to the results described above, ES induced a similar effect on Ca^2+^ handling as did K^+^-induced membrane depolarization on ΔΨmt. ES reduced ΔΨmt in the SSM region with a maximal effect at 35s (±4 s), and this reduction was reversed after 10 min (Figure 6B, left panel). Both, ES and high K^+^ induced similar maximal effects on ΔΨmt in the SMM; the drop of ΔΨmt was −47% ± 8.3 and −50% ± 12.3 for ES and high K^+^, respectively (Figure 6B, right panel). Importantly, high K^+^ medium induced a transient ΔΨmt drop in SSM (Figure S4), indicating that this membrane depolarization strategy does not damage mitochondrial function.

Interestingly, the ΔΨmt reduction in the subsarcolemmal region observed after cell membrane depolarization -with either ES or high potassium medium- was partly prevented with either xestospongin B or dantrolene, without difference in the slope of mitochondrial depolarization. The reduction of ΔΨmt was completely abolished when both inhibitors were used (Figures 6C,D), suggesting that both IP_3_R and RyR1 participate in ΔΨmt reduction in the subsarcolemmal region after membrane depolarization, and indicating that mitochondrial depolarization is not an indication of a permanent damage of mitochondria caused by K^+^ solution but rather resulted from a physiological Ca^2+^-dependent mechanism. Additionally, membrane depolarization modified differently ΔΨmt in the SSM compared to the IMFM. As shown in Figures 6E,F, membrane depolarization induced a decrease in ΔΨmt, which was particularly evident in the SSM, while in the IMFM ΔΨmt showed an increase. This ΔΨmt increase in the IMFM occurred subsequent to the depolarization of the SSM. The mitochondrial membrane potential reduction in SSM correlated with a mitochondrial Ca^2+^ increase in this area (Figure 6E). Although we cannot rule out an early decrease in IMFM ΔΨmt upon the Ca^2+^ increase, this change was not evident.

These results suggest that there is transfer of ΔΨmt from the SSM to the IMFM; presumably this transfer takes place after depolarization by a Ca^2+^-dependent mechanism.

## Discussion

### Role of the intracellular calcium channels on mitochondrial calcium increase after membrane depolarization

The mitochondrial network in the muscle fiber exhibits a particular architecture in which a large portion of mitochondria resides in close proximity to myofibrils where ATP production is essential for contraction; these mitochondria are known as intermyofibrillar mitochondria or IMFM (Picard et al., 2013). The sub-sarcolemmal mitochondria or SSM is a second type of mitochondria (also called perivascular mitochondria); their location, function and spatial organization are quite different to the IMFM (Picard et al., 2013; Glancy et al., 2015). Interestingly, a continuum of mitochondria is present through the inner surface of the muscle fiber, up to a depth of several micrometers, and it is particularly dense around the nuclei (Glancy et al., 2015). Our observations and previous reports in striated muscle (Glancy et al., 2015, 2017; Lavorato et al., 2017) suggest that in adult skeletal muscle fibers the mitochondria constitute a semi-permanent extensive network that allows intracellular energy distribution. Thus, SSM and IMFM could work together as one organelle in order to satisfy the bioenergetics requirements of the adult muscle fiber.

Although a mitochondrial calcium increase after depolarization has been already studied in isolated adult muscle fibers using electrical stimulation or high potassium medium (Casas et al., 2010; Mammucari et al., 2015), the role of intracellular Ca^2+^ channels in this response is poorly understood. In this work, the mitochondrial Ca^2+^ increase after depolarization was partly mediated by both RyR1 and IP_3_R. Other authors have reported that Ca^2+^ release mediated by IP_3_R does not occur in adult skeletal muscle fibers (Blaauw et al., 2012). There are several reasons why such signals may be hard to record, including the recently predicted small range of action of IP_3_ (< 5μm) (Dickinson et al., 2016), the functional distribution of IP_3_R inside the cell (Dickinson et al., 2016) and mitochondrial buffering of the Ca^2+^ transient (Eisner et al., 2010). In this work, we showed that the mitochondrial Ca^2+^ increase after depolarization was partly prevented by IP_3_R inhibition (xestospongin B) or downregulation (shIP_3_R-RFP), and that locally photo-released IP_3_ increased mitochondrial Ca^2+^ levels in 50% of the cells evaluated. Based on these combined results we strongly suggest that both RyR1 and IP_3_R mediate the mitochondria Ca^2+^ increase detected after depolarization of adult skeletal muscle fibers.

It is interesting to note that about half of the mitochondrial calcium transient appears to be due to the activity of RyR1 channels, and the other half is due to IP_3_R activity, despite the large difference in the amplitude of these two cytosolic signals. The explanation appears to be the differential location of these receptors. In fact, both type-1 and type-2 IP_3_R have been described to be preferentially located on the surface of the fiber (Casas et al., 2010; Tjondrokoesoemo et al., 2013), while RyR1 channels are preferentially located at the triad (Franzini-Armstrong, 2009), present in the intermyofibrilar region. Previous studies reported that there is functional and structural communication between mitochondria and SER (Rizzuto and Pozzan, 2006; Bravo-Sagua et al., 2013). In several cells lines, the IP_3_R located in the SER is close to the voltage-dependent anion channel in the outer mitochondrial membrane, generating a microdomain where intracellular Ca^2+^ transference from SER to mitochondria has been postulated to take place (Rizzuto et al., 1998; Rizzuto and Pozzan, 2006).

We can speculate then that about half of the mitochondria in this particular muscle fiber will be located in the subsarcolemmal region, in close contact with SER that contains IP_3_R channels and the other half of the mitochondria will be close to the triad, rich in RyR channels. Whilst RyR-mediated Ca^2+^ release to the cytoplasm could reach the mitochondrial matrix (probably of both populations) because of the high concentration reached by this ion in the cytosol, the direct transference of Ca^2+^ from SER to the mitochondria through the IP_3_R channel might explain why both RyR1 and IP_3_R channels contribute in a similar magnitude to the increase of mitochondrial Ca^2+^ level after depolarization. Furthermore, the two mitochondria populations appear to have a differential content of proteins (MICU1) needed for calcium entry regulation.

In this work, we used the mitochondria Ca^2+^ sensor Cepia3*mt* (Suzuki et al., 2014). Until now this tool has not been used to evaluate mitochondrial Ca^2+^ handling in adult skeletal muscle fibers. Cepia3*mt* (Kd 11 μM) exhibited significant preferential mitochondria localization (evaluated through its co-localization with mtDsRed). It was previously reported that in muscle fibers the maximal mitochondrial Ca^2+^ increase reached during a prolonged depolarization pulse exhibits a small delay compared to the cytoplasmic Ca^2+^ transient (Karam et al., 2017). Nevertheless, the kinetics of Cepia3*mt* compared to other mitochondrial Ca^2+^ sensors appears to be even slower (Rudolf et al., 2004; Karam et al., 2017); this might be explained by Cepia3*mt* characteristics, other than its Kd (Suzuki et al., 2014).

### Role of the intracellular calcium channels on OCR increase after membrane depolarization

The main mechanism proposed to explain Ca^2+^ regulation of mitochondrial metabolism, suggest that matrix Ca^2+^ accumulation stimulates several enzymes of the Krebs cycle (Das and Harris, 1990; Denton, 2009), thus providing increased NADH to foster oxidative phosphorylation and in consequence augment the mitochondrial OCR and ATP synthesis. Several experimental pieces of evidence from this study point in this direction. First, there is indeed an increase in basal O_2_ consumption and in ATP synthesis-dependent O_2_ consumption minutes after depolarization of isolated skeletal muscle fibers. Second, mitochondrial Ca^2+^ increases after either electrical stimulation or K^+^-induced depolarization. Third, in the resting condition, the basal OCR and ATP-linked OCR were partly dependent on RyR1 and partly on IP_3_R. Fourth, the ATP synthesis-dependent OCR increase produced by depolarization appears to be dependent on Ca^2+^ release from RyR1.

Notwithstanding the fact that RyR1 and IP_3_R participate in the mitochondrial Ca^2+^ increase produced by depolarization, only RyR1 mediated the increased OCR after depolarization, suggesting a critical role of RyR1 on mitochondrial metabolism during the excitation-contraction coupling cycles. In agreement with our observations, Brieni et al. overexpressing a mutated RyR1 associated with malignant hyperthermia (RyR1-MH) in HEK-293 cells reported that RyR1-MH enhanced mitochondrial Ca^2+^ levels (Brini et al., 2005). In addition, skeletal muscle mitochondria from heterozygous mice expressing the human MH/CCD RyR1 R163C mutation exhibited a higher level of matrix Ca^2+^ compared with the wild type (Boncompagni et al., 2009). Considering that the ability of the mitochondria to take up Ca^2+^ depends partly on the magnitude and duration of the cytoplasm Ca^2+^ transient (Wacquier et al., 2016), we speculate that after depolarization, the huge and fast Ca^2+^ transient mediated by RyR1 could be enough to satisfy the mitochondrial Ca^2+^ increase required to enhance mitochondrial metabolism and O_2_ consumption. However, the physiological role of the IP_3_R-dependent mitochondrial Ca^2+^ increase in adult skeletal muscle fibers remains to be explored. In muscle fibers, an interaction between SER and mitochondria has been proposed to rely on privileged microdomains where Ca^2+^ is readily transferred during excitation-contraction coupling from SER to mitochondria (Pietrangelo et al., 2015). It is possible then that IP_3_R and RyR1 are differentially important for mitochondria residing on different locations of the fiber and that these two populations of mitochondria have different, although complementary functions.

Despite that both intracellular Ca^2+^ channels regulate the mitochondrial OCR, it is still unknown if this regulation occurs by mitochondrial matrix Ca^2+^ accumulation or by a Ca^2+^ increase in other compartments, such as the intermembrane space or the cytoplasm. It is important to consider that RyR1 inhibition completely prevented the increase of OCR after depolarization, even when the mitochondrial Ca^2+^ increase was only partly inhibited. One possibility is that massive Ca^2+^ release through RyR1 channels could activate different Ca^2+^-sensitive enzymes, such as the Sarcoplasmic/endoplasmic reticulum calcium ATPase 1 (SERCA1) (Murray et al., 1998) resulting in increased ATP utilization. ATP degradation is driven to intermediate metabolites accumulation that may go into the mitochondria matrix, activating the Krebs cycle and increasing OCR. Moreover, Ca^2+^ transients activate different transporter proteins in the inner mitochondrial membrane, such as the aspartate/glutamate exchanger (Pardo et al., 2006). Whilst the Ca^2+^ affinity of intra-mitochondrial dehydrogenases is in the micromolar range (Denton et al., 1972), the inner mitochondrial membrane transporter requires a lower Ca^2+^ increase. For instance, the aspartate/glutamate exchanger presents an EC50 for Ca^2+^ of 324 ± 57 nM (Pardo et al., 2006). Additionally, computational simulations have suggested the existence of parallel mechanisms (intramitochondrial and cytoplasmatic mechanisms) that would regulate the mitochondrial function (Korzeniewski, 2007). A dynamic equilibrium among the different processes regulated by Ca^2+^ transients in order to regulate mitochondrial function is probably required.

### Heterogeneity of mitochondrial populations in adult skeletal muscle fibers

The SSM in their location, function and spatial organization are quite different to the IMFM (Picard et al., 2013; Glancy et al., 2015). For instance, using direct measurement of superoxide by electron paramagnetic resonance, Crochemore et al. (Crochemore et al., 2015) showed that SSM produce more superoxide than IMFM. It has been shown recently that electron transport chain elements (ETC) are differentially distributed among SSM and IMFM; in particular, there are differences in the distribution of complex V (ATP synthase) and Complex IV (Glancy et al., 2015). In line with these results, in this study we observed that cytochrome C, another component of the ETC, is located mostly in the SSM. In contrast, the importin TOM20 has homogeneous distribution into the cell, suggesting a specific compartmentalization of different mitochondrial proteins between mitochondria subpopulations. An important open question is how SSM and IMFM are functionally coupled. Previous reports based on structural data and local mitochondrial uncoupling suggested that the SSM and IMFM work as a syncytium with regard to the proton-motive force (ΔΨ + *pH* gradient across the inner mitochondrial membrane), thus the ΔΨmt may be distributed toward IMFM to generate ATP in the intermyofibrillar region (Glancy et al., 2015). Additionally, the analysis of the mitochondrial network in human skeletal muscle by SEM and 3D reconstructions shows clear continuous tubular connections between IMF mitochondria and SS mitochondria (Dahl et al., 2015). Our experiments suggest a slow, calcium-dependent mechanism of transfer of ΔΨmt between the SSM and IMFM that might activate ATP synthesis. The lines of evidence that suggest a functional interaction between the mitochondria subpopulations presented in this study are: First; The intramitochondrial protein distribution in the SSM substantially differs from IMFM; second, after depolarization, ΔΨmt decreases in SSM to later increase in the IMFM in a way compatible with a propagation process from one region to another, and this ΔΨmt change correlates with the mitochondrial calcium increase. We have shown direct evidence linking mitochondrial calcium signals with changes in mitochondrial membrane potential and the kinetics of the former always precede the changes in ΔΨmt. Because of the slow kinetics of ΔΨmt change from one mitochondrial subpopulation to the other, we speculate that either an unknown “switch” activates communication at a given time, or a diffusion process takes place within the mitochondria network that may explain the functional connection between SSM and IMFM. A mechanism was proposed by Skulachev (2001), in which protons can travel along mitochondria from the surface to intermyofibrilar regions. As protons are likely to be bound to the lipids in the mitochondria membrane (Xu et al., 2016), it is tempting to speculate that calcium ions could displace protons from such binding sites thus favoring diffusion. On the other hand, recently Patel et al., proposed a spreading mechanism driven by diffusion of K^+^ and/or Na^+^ ions (Patel et al., 2016). The fact that IP_3_R and RyR1 participate differentially in OCR changes after membrane depolarization may suggest a different role of calcium signals either inducing SSM mitochondria depolarization or a Ca^2+^ dependent IMFM ΔΨmt (and OCR) increase. In fact, the calcium increase in the SSM is larger and frequently precedes the calcium changes in IMFM, possibly due to differential mitochondrial protein distribution; this may be influencing the effect of Ca^2+^ over ΔΨmt in these two regions.

Mitochondria depolarization after ES depends on both intracellular Ca^2+^ channels, suggesting a Ca^2+^-dependent mitochondria depolarization after ES. Furthermore, depolarization appears to occur preferentially in the subsarcolemmal region and an increase in mitochondrial membrane potential is evident in intermyofibrilar regions, while oxygen consumption, in particular that one linked to ATP synthesis, appears to be sensitive to RyR1 inhibition. We can speculate that after membrane depolarization, calcium signals trigger the spreading of ΔΨmt toward intermyofibrilar mitochondria, enhancing ATP synthesis-dependent O_2_ consumption. This ΔΨmt spread would explain why blockade of IP_3_R affects exclusively the basal levels of O_2_ consumption while RyR blockade decreases the component activated by depolarization, and in particular the one dependent on ATP synthesis. However, we cannot discard the participation of some extra-mitochondrial mechanisms that might regulate mitochondrial function in a Ca^2+^-dependent fashion.

An increase in ΔΨmt in mitochondria that are partly depolarized will immediately increase ATP production, especially in those particular mitochondria that express large amounts of ATP synthase. In resting conditions, ATP production in skeletal muscle is relatively low but it increases more than two orders of magnitude in exercise conditions (Weibel and Hoppeler, 2005); the mechanism that we are visualizing here will allow this phenomenon to proceed and will comply with the immediate muscle fiber energy requirements. We named this process “excitation-metabolism coupling” because it establishes a connection between membrane potential depolarization, normally occurring with the skeletal muscle action potential, and the large metabolic increase that has been related to exercise and muscle contraction.

## Materials and methods

### Adult skeletal muscle fibers isolation

All experiments were carried out in accordance with protocols approved by the Animal Care Committee at the University of Chile and were consistent with ARRIVE guidelines. For all experiments, *Flexor digitorium brevis* (FBD) muscle was obtained from C57BL6/J mice between 8 and 10 weeks of age as previously described (Díaz-Vegas et al., 2015). Briefly, muscle tissue was dissected and incubated with collagenase type IV (2.7 mg/mL) (Worthington, Lakewood, NJ) during 90 min at 37°C followed by mechanical dissociation with polished pipettes. Isolated muscle fibers were plated in the extracellular matrix (ECM) gel-coated (Sigma-Aldrich, E1270) in DMEM supplemented with 10% horse serum, 50 U/mL penicillin, 50 mg/mL streptomycin. After overnight incubation, muscle fibers were used for experimentation.

### Electrical stimulation protocol

Isolated muscle fibers were exposed to electric field stimulation (ES). The ES protocol was a single sequence of 270 square pulses of 0.3 ms duration at 20 Hz or 90 Hz (lasting 13.5 or 3 s) with platinum electrodes wires intercalated 0.5 cm apart, as described previously (Jorquera et al., 2013).

### Plasmid construct

The following plasmids were used. pCMV-Cepia3mt (Invitrogen N° 58219), pCMV- RCamP 1.07 (RCamPs) (plasmid map is shown in Figure S6) and shIP_3_R1-pRFP –C-RS (Origene TF517036).

### Electroporation protocol

Electroporation protocol was performed as previously reported (DiFranco et al., 2009). Briefly, anesthetized mice were injected with hyaluronidase (10 μL of 2 mg/mL) dissolved in sterile saline at the ventral side of the hind paws through a 29-gauge needle. 60 min later, 5–10 μg/μL of suitable plasmid DNA in 10 μl of sterile saline was injected into the same sites. Fifteen minutes later, two electrodes (gold-plated stainless steel acupuncture needles) were placed at the starting lines of paw and toes, separated by about 9 mm. 20 pulses of 100 V/cm with 20 ms duration were applied at 1 Hz. Seven days later, the mice were euthanized and FDB muscles were removed for functional studies.

### Oxygen consumption rate

Oxygen Consumption Rate (OCR) was evaluated using XF96 Seahorse Extracellular Flux Analyzer (Seahorse Agilent Technologies ©). Fibers obtained from 4 mice were plated 12 h before the experiment, in Seahorse XF^e^ of 96 wells the average of these fibers were considered as one experiment; 4 independent experiments were performed in total. In order to remove the dead muscle fibers, each well was washed once with Seahorse medium before the stimulation protocol. OCR evaluation was performed with Seahorse XF^e^ Cell Mito Stress Test kit according to manufacturer's protocol. Muscle fibers were incubated at 37°C without CO_2_ during 2 h in control Seahorse XF Assay Medium containing 1 mM glutamine, 1 mM pyruvate, 10 mM glucose, 145 mM NaCl, pH 7.4. Before the start of the OCR test muscle fibers were stimulated with isotonic high K^+^ (65 mM) Seahorse medium or control isotonic Seahorse medium during 1 min. Then, each well was washed with control seahorse medium and the plate was incubated for 5 min. At the end of this time, OCR was evaluated. To analyze mitochondrial function, fibers were treated sequentially with oligomycin (10 mg mL^−1^), carbonyl cyanide-p-trifluoromethoxyphenylhydrazone (FCCP, 500 nM), and rotenone (1 μM). OCR was recorded following each injection. Each experimental condition was assigned to 16 wells per plate (control, Xest. B, Dantro, K^+^, Xest. B plus K^+^ or Dantro. Plus K^+^). The OCR value of each well was normalized against its own protein concentration. We excluded from the analysis the wells that contained less than 1 μg of protein, corresponding to 30% of the total wells evaluated by each condition. In order to avoid the contamination with myoblasts, the purity of the muscle fibers culture was over 95%.

The protein concentration in each condition was: control 0.135 μg/μL (±0.02); Xesto. B 0.158 μg/μL (±0.02); Dantro. 0.16 μg/μL (±0.02); K^+^ 0.206 μg/μL (±0.04); K^+^ + Xesto. B 0.186 μg/μL (±0.04); K^+^ + Dantro. 0.119 μg/μL (±0.02). The protein concentration of each well was determined immediately after de OCR assay.

### Real-time imaging

All experiments were conducted in Krebs Ringer buffer (in mM): 145 NaCl, 5 KCl, 1 CaCl_2_, 1 MgCl_2_, 5.6 glucose, 10 HEPES, pH 7.4 at room temperature in the presence of 75 μM N-benzyl-P-toluenesulfonamide (BTS, Sigma- Aldrich) to inhibit muscle contraction. The real-time imaging experiments were performed by confocal microscopy using Carl Zeiss Pascal 5, LSM with a PlanApo 64 × /1.4 N.A. oil immersion objective, Leica TCS-SP5-II with a 100 × /1.4 N.A. Plan-apochromat objective equipped with STED system. The pixel size was set below 100 nm to meet the Nyquist criterion. Pinhole size was set at 1 Airy unit and z-stacks were acquired with a step size of 130 nm. Lateral and axial resolutions were approximately 230 and 460 nm, respectively; determinations were done in an inverted Olympus IX81 microscope with a 40 × /N.A. 1.3 oil immersion objective or in a spinning disk microscope (PerkinElmer, Waltham, MA/Zeiss, Oberkochen, Germany) Plan-NEOFLUAR 100 × /1.3 N.A. oil immersion objective. Muscle depolarization was performed using the isotonic high K^+^ medium (65 mM) or ES.

### Mitochondrial Ca^2+^ measurement

Mitochondrial Ca^2+^ measurement was performed 7 days after *in vivo* electroporation with plasmids for expression of either Cepia3*mt* or mtGcaMP6m. The electroporation was performed according to previously reported procedures (DiFranco et al., 2009). Mitochondrial Ca^2+^ levels were evaluated with Mitochondrial-targeted Cepia3*mt*. Cepia3*mt* fluorescence was obtained using the excitation-emission at λ488/λ510–540 nm, the laser gain was kept at 4%, the images acquired were every 1 s in the Carl Zeiss Pascal 5 and the Leica TCS-SP5-II microscope for *x,y* and *x,z* time lapse respectively. The *x,y*-axis time-lapse experiments in the TCS-SP5-II microscope were performed every 250 ms. After 50 s of basal line acquisition, high K^+^ solution or ES were applied. Changes in Ca^2+^ levels were expressed as (ΔF/F_0_)^*^100. Mitochondrial Ca^2+^ level at resting condition was evaluated by measuring mtGcaMP6m fluorescence in the inverted Olympus IX81 microscope. Seven days after electroporation muscle fibers were isolated and mtGcaMP6 fluorescence was detected using an excitation/emission wavelength λexc1–λexc2/λem = 400–490/520 nm. The ratio between the signals excited with 490 and 400 nm was used to determine the Ca^2+^ level. Noise in the images was corrected frame by frame by subtracting the mean pixel value of a cell-free region of interest (ROI) using Fiji distribution of ImageJ (Schindelin et al., 2012). The ROI into the cell was determined after the projection of the standard deviation of fluorescence using Fiji (Image/Stack/*z* Project/Standard Deviation); for analysis 20 ROI were selected per fiber.

### Cytosolic Ca^2+^ measurements

Muscle fibers were isolated 7 days after *in vivo* electroporation with the cytosolic Ca^2+^ indicator red GECI (RCamPs) (Akerboom et al., 2013). RCamPs fluorescence was detected using the excitation-emission wavelength at λ545-λ580/590 nm, keeping the laser gain at 4%, the images were acquired every 1s in the Carl Zeiss Pascal 5 microscope in *x,y*-axis scan mode. After 50 s of basal line acquisition, ES was applied. Changes in Ca^2+^ levels were expressed as (ΔF/F_0_)^*^100. The background was corrected against the mean pixel value of a cell-free ROI using Fiji. During experiments, the fibers were maintained in Krebs buffer and images were acquired every 1 s. For the analysis 20 ROI were selected per cell.

### ΔΨmt measurements

ΔΨmt was measured by loading fibers with 20 nM tetramethylrhodamine, ethyl ester (TMRE^+^, Life Technologies) for 40 min at 37°C plus mitotracker green (MTG). MTG was used to normalize the fluorescence among the different mitochondrial populations. TMRE^+^ fluorescence was detected using the excitation-emission λ545–580/590 nm using Carl Zeiss Pascal 5 microscope. For kinetic determinations, images were acquired every 1 s with 1% laser gain maintained under 1%, in order to avoid quenching and fluorescence toxicities. FCCP (0.5 μM) or Oligomycin (1 μM) was added at the end of the experiment to blunt-collapse ΔΨmt. The ΔΨ*mt* was evaluated as raw fluorescence intensity of background-corrected images by subtracting the fluorescence intensity mean value after FCCP or oligomycin administration from the average value of the first 10 acquisitions (ΔF).

### Photorelease of caged IP_3_

Muscle fibers were electroporated with a plasmid for expression of Cepia3*mt*. Seven days after, skeletal muscle fibers were isolated and incubated with the membrane permeant form of IP_3_ (5 μM, D-2-3-*O*-isopropylidene-6-*O*-(2-nitro-4,5-dimethoxy)benzyl-myo-inositol-triphosphate-hexakis(propionoxymethyl) ester; Sichem GmbH) during 45 min at 37°C. Fibers were placed in the microscope under the beam of the UV laser (Micropoint laser, Andor, Belfast, Northern Ireland). After acquiring a 20 s baseline at 10 Hz, photorelease of caged IP_3_ was performed by applying a 435 nm UV laser at 15 Hz to a mitochondrial local spot (~3–4 μm in diameter). Fluorescence signals were recorded at 10 Hz in a spinning disk microscope (PerkinElmer, Waltham, MA/Zeiss, Oberkochen, Germany). The results were processed with the formula (ΔF/F0)^*^100. The background signal recorded in cell-free regions was used to correct the fluorescence.

### Immunofluorescence

The immunofluorescence analysis was performed according to the previous report with modifications (Mammucari et al., 2015). Briefly, FDB fibers were rinsed with ice-cold PBS, fixed in 4% paraformaldehyde in PBS for 15 min (Electron Microscopy Science, Hatfield, PA, USA) and quenched with 50 mM NH_4_Cl in PBS for 10 min. Cells were washed with ice-cold PBS and permeabilized for 20 min with 1% Triton X-100 in PBS and blocked in PBS-BSA 4% (w/v) during 45 min. Fibers were incubated in PBS-BSA 2% (w/v) with antibody against TOM20 (Santa Cruz, Rabbit) 1:100; Cytochrome C (Bioscience, Mouse) 1:100, complex IV (Subunit I, Life Technologies, Mouse) 1:100 and MCU (Sigma, Rabbit) 1:50, MICU1 (Sigma, Rabbit) 1:50 and anti-subunit alpha of the ATPase (ABCAM, mouse) 1:50 overnight at 4°C. Finally, cells were washed three times with PBS during 5 min each and incubated with secondary antibody anti-mouse and anti-rabbit Alexa Fluor 488/Alexa Fluor 546 as appropriate and coverslips were mounted with ProLong Gold Antifade reagent (Life Technologies). Images were taken in both *x,y* and *x,z*-axis scan using the Leica TCS-SP5-II microscope equipped with 40x, 1.25 N.A., Plan-apochromat objective, in STED configuration. 488 nm and 543 nm laser lines and images were collected in the 496–550 nm and 580–690 nm ranges. The pinhole was set to 1.0 airy units and pixel size was set to 48.88 nm. Image deconvolution and processing were performed using Fiji distribution of ImageJ. Mander's coefficients were calculated using the JACoP plugin for Fiji (Bolte and Cordelieres, 2006).

### Western blot

One week after electroporation with shIP_3_R1-RFP or Scrambled-RFP, FDB muscles were isolated and homogenized using an electric homogenizer (Fluko, Shanghai, China) in a lysis buffer containing in mM: 20 Tris-HCl (pH 7.5), 1% Triton X-100, 2 EDTA, 20 NaF, 1 Na2P2O7, 10% glycerol, 150 NaCl, 10 Na3VO4, 1 PMSF and protease inhibitors (Complete™, Roche Applied Science). Protein separation was performed using SDS-PAGE followed by transfer to PVDF membranes. The following primary antibodies were used: anti-IP_3_R1 and horseradish peroxidase-conjugated secondary antibodies (Santa Cruz Biotechnology, CA, U.S.A.). The protein bands in the blots were visualized using a WESTAR Supernova detection kit (Cyanagen, Bologna, Italy) and ChemiDoc™ MP System (Bio- Rad, USA). The intensity of the bands was determined by ImageJ densitometry analysis.

### Data analysis

All experimental results are expressed as means ± S.D. For paired data and for multiple comparisons we used Student's *t*-test and one-way ANOVA followed by Dunnett's post-test respectively. For multiple paired comparisons, One-way ANOVA followed by Bonferroni's post-test was performed. *P* < 0.05 was considered statistically significant. Graph-Pad PRISM version 5.0 software (GraphPad Software Inc, LA, USA) was used for data fitting.

## Author contributions

AD-V designed and performed the majority of the experiments; he wrote and discussed the manuscript. AC performed and analyzed the photo-release of IP3 experiments. DV discussed and wrote part of the manuscript; she participated in the data analysis. PL performed knock-down of IP3R experiments. CH contributed in data discussion, writing and discussion of the manuscript. GG performed and analyzed MEF-cells KO for MICU1; she collaborated in immunofluorescence measurements of intramitochondrial proteins. DD participated in data discussion, writing and discussion of the manuscript. CM participated in data discussion, writing and discussion of the manuscript. RR participated in data discussion, writing and discussion of the manuscript. AC-F participated in the main idea of the manuscript, design of experiments, data analysis and discussion of the manuscript. EJ participated in planning, design of experiments and the main idea of the manuscript, data discussion and manuscript writing.

### Conflict of interest statement

The authors declare that the research was conducted in the absence of any commercial or financial relationships that could be construed as a potential conflict of interest.
