# Supplementary material for: Mitochondrial Calcium Increase Induced by RyR1 and IP3R Channel Activation After Membrane Depolarization Regulates Skeletal Muscle Metabolism
Source: Front Physiol. 2018 Jun 25;9:791. doi: 10.3389/fphys.2018.00791 (PMC6026899; doi:10.3389/fphys.2018.00791)
Supplement: Supplementary file 9 [file Data_Sheet_1.docx]

Supplementary Materials

6.1 Knockdown of IP_3_R1 and Western Blot

One week after electroporation with shIP_3_R1-RFP or Scrambled-RFP, FDB muscles were isolated and homogenized using an electric homogenizer (Fluko, Shanghai, China) in a lysis buffer containing in mM: 20Tris-HCl (pH 7.5), 1% Triton X-100, 2 EDTA, 20 NaF, 1 Na2P2O7, 10% glycerol, 150 NaCl, 10 Na3VO4 , 1 PMSF and protease inhibitors (Complete ^TM^, Roche Applied Science). The separation of the protein was performed using SDS-PAGE followed by transfer to PVDF membranes. The following primary antibodies and their dilutions were used: anti-IP_3_R1 and horseradish peroxidase-conjugated secondary antibodies (Santa Cruz Biotechnology, CA, U.S.A.). The protein bands in the blots were visualized using a WESTAR Supernova detection kit (Cyanagen, Bologna, Italy) and ChemiDoc^TM^ MP System (Bio- Rad, USA). The intensity of the bands was determined with ImageJ densitometry analysis.

6.2 Muscle fiber permeabilization

Adult muscle fibers were electroporated with plasmids encoding Cepia3*mt*. 7 to 10 days after electroporation the fibers were isolated and permeabilized with saponin (50 μg ml^−1^) during 40 s and then immersed into one of the ‘internal’ solutions as previously reported ^58^. Internal solutions contained (in mM): (in mM): 140 potassium glutamate, 5 Na_2_-ATP, 5 sodium phosphocreatine, 5.5 MgCl_2_, 5 D-glucose, 0.1 EGTA-AM, 5 HEPES, adjusted to pH 7.2 with KOH. After permeabilization, muscle fibers were incubated during 30 min with internal solution or internal solution plus Ruthenium Red (1 μM). After incubation the muscle fibers were stimulated with 10 μM or 30 μM CaCl_2_.

**Supplementary Figures**

**
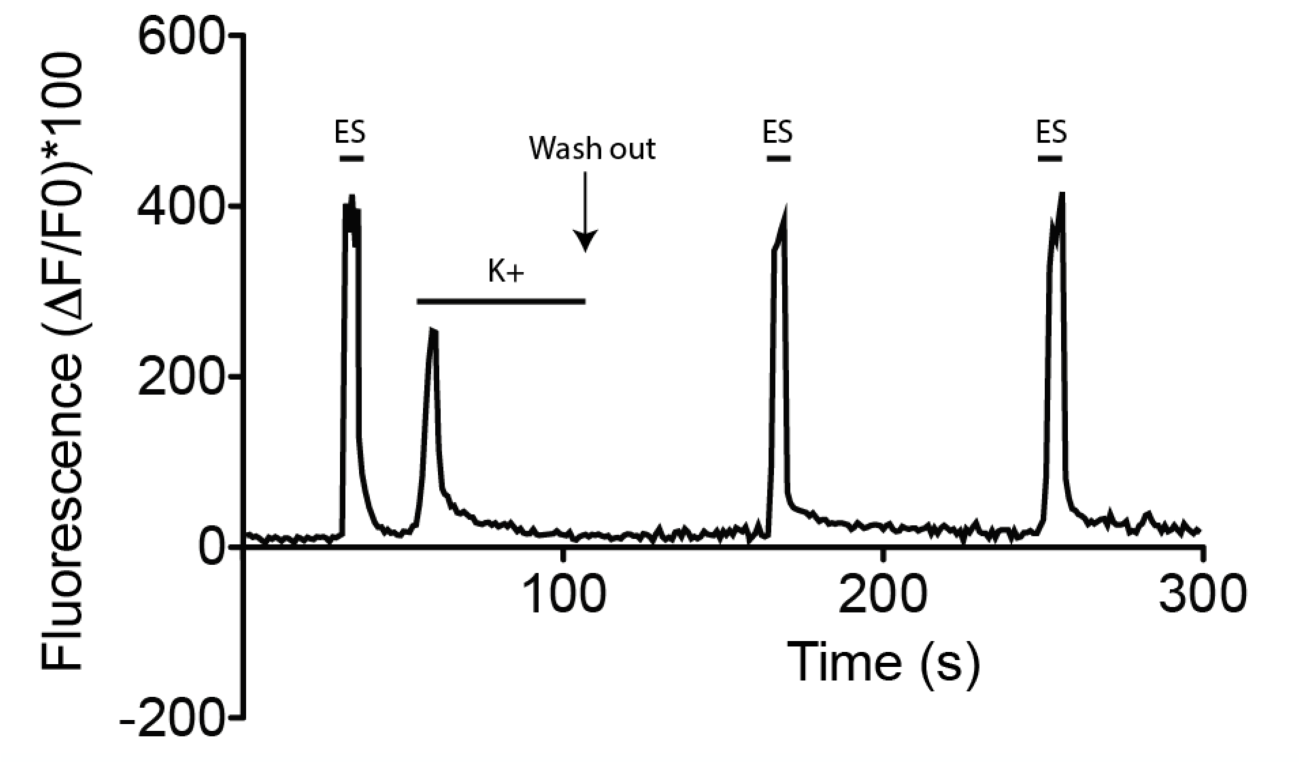
**

**Fig. S1. High K^+^ medium does not affect cell membrane excitability.** FDB muscle fibers were loaded with Fluo4-AM 30 min before the start of the experiment. The muscle fibers were maintained in isotonic Krebs buffer and subjected to ES or isotonic high potassium medium (65mM). 40 sec after potassium stimulation the medium was washed and replaced by isotonic Krebs solution (arrow).

**Figure 2**

**Fig. S2. Knock down of IP_3_R1 in adult skeletal muscle fiber.** FDB muscle was electroporated with plasmid encoding either shIP_3_R1-RFP or Luc-RFP and one week later the levels of IP_3_R1 were evaluated. Data were normalized against GAPDH level. N=3 different animals, *** p<0.001

**Figure 3**

*
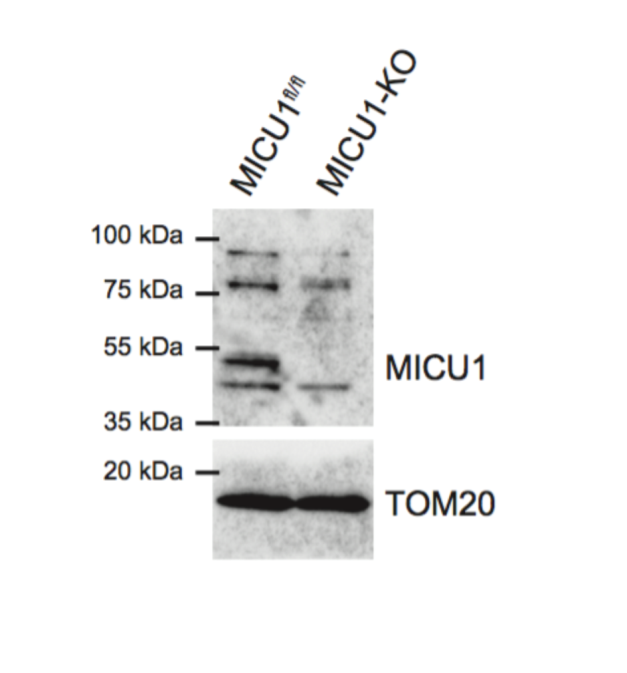
*

**Fig. S3. MICU1 antibody validation.** The MICU1 antibody was validated in whole lysates of WT or MICU1 KO cells by western blot.

**Figure 4**

**
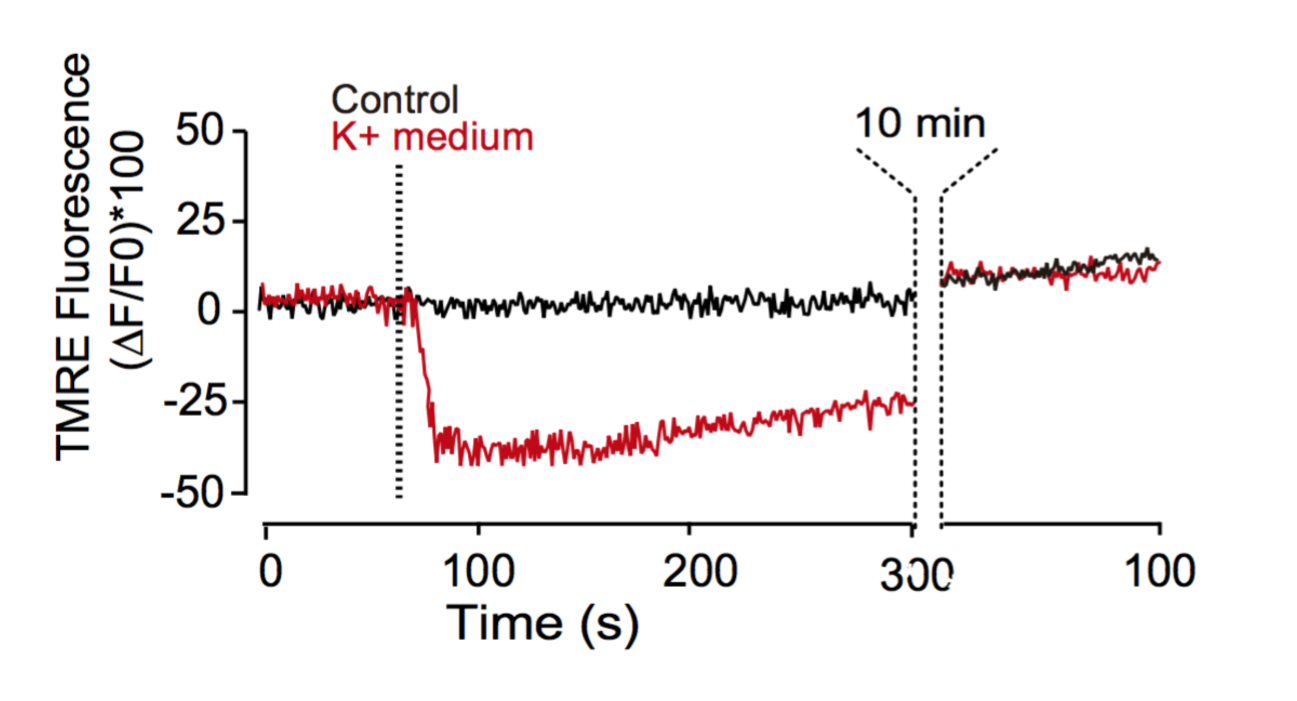
**

**Fig. S4. High K^+^ medium induced a transient depolarization of SS mitochondria.** FDB muscle loaded with TMRE^+^ 30 min before the start of the experiment. The muscle fibers were maintained in isotonic Krebs buffer and exposed to isotonic high potassium medium (65 mM). 40 s after potassium stimulation the medium was replaced by isotonic Krebs solution.

**Figure 5**

**Fig. S5. Ruthenium Red prevented mitochondrial Ca^2+^ increase.** Muscle fibers were electroporated with plasmids encoding CEPIA3mt and permeabilized with 0.004% of saponin during 40 s. Extracellular Ca^2+^ pulses were applied (arrow) in control fibers or in fibers pre-incubated with ruthenium red. Representative kinetic records of 6 different fibers.

**Figure 6**

**Fig. S6.**  **Plasmid map of RCamPs provided by Dr. M Ohkura from Saitama University**

**7. Supplementary videos**

**7.1 Movie S1. Mitochondrial Ca^2+^ increased after depolarization using CEPIA3mt in skeletal muscle fibers.** *In vivo* electroporation of FDB muscle was performed and one week later muscle fibers were isolated and mitochondrial Ca^2+^ levels were determined. The video shows a crop of the muscle fiber. The change of fluorescence is shown in pseudo-color.

7.2 Movie S2. Intramitochondrial Ca^2+^ wave-like propagation in the subsarcolemmal region of the skeletal muscle fibers. The experimental protocol was performed according to *movie 1.*

7.3 Movie S3. SSM and IMFM Ca^2+^ levels after depolarization. *In vivo* electroporation of FDB muscle was performed and one week later muscle fibers were isolated. Mitochondrial Ca^2+^ levels were determined after depolarization in *y,z,x* axis. The change of fluorescence is shown in pseudo-color.

7.4 Movie S4. Cytochrome C is enriched in the SSM compared to IMFM. FDB muscle fibers were isolated and immunofluorescence against cytochrome C was performed. The threshold was determined using Image J for positive label of cytochrome C and several slices were analyzed.

7.5 Movie S5. MCU has a homogeneous distribution in both SSM and IMFM in the muscle fiber. FDB muscle fibers were isolated and immunofluorescence against cytochrome C (red) and MCU (green) was performed. Co-localization of both proteins is shown (merge, yellow). 3D reconstruction of 30 µm was carried out.

7.6 Movie S6. MICU1 has a distribution restricted to IMFM in the muscle fiber. FDB muscle fibers were isolated and immunofluorescence against cytochrome C (red) and MICU1 (green) was performed. Co-localization of both proteins is shown (merge, yellow). 3D reconstruction of 30µm was carried out.

7.7 Movie S7 and Movie S8. Depolarization increased the mitochondrial Ca^2+^ level (S7) and reduced the mitochondrial membrane potential (S8) in the subsarcolemmal region. *In vivo* electroporation of FDB muscle was performed and one week later muscle fibers were isolated and loaded with TMRE^+^ in non-quenching mode (20nM). A subsarcolemmal optical slice is shown. Mitochondrial Ca^2+^ levels (Magenta-yellow, S7) and Δ*Ψ*mt (Orange-Yellow, S8) were determined after depolarization. The change of fluorescence is shown in pseudo-color. 300 s of recording and each photo was collected every second. The high K^+^ solution was added at 50 sec. FCCP (0.5 μM) was added at 230 s.
